# Self-Healable Covalently Adaptable Networks Based on Disulfide Exchange

**DOI:** 10.3390/polym14193953

**Published:** 2022-09-21

**Authors:** Xinru Guo, Feng Liu, Meng Lv, Fengbiao Chen, Fei Gao, Zhenhua Xiong, Xuejiao Chen, Liang Shen, Faman Lin, Xuelang Gao

**Affiliations:** 1 Jiangxi Engineering Laboratory of Waterborne Coating, School of Chemistry and Chemical Engineering, Jiangxi Science & Technology Normal University, Nanchang 330013, China; 2 Department of Chemistry, Pohang University of Science and Technology (POSTECH), Pohang 790-784, Korea

**Keywords:** self-healing, vinylogous urethane, disulfide bonds, activation energy

## Abstract

Introducing dynamic covalent bonding into thermoset polymers has received considerable attention because they can repair or recover when damaged, thereby minimizing waste and extending the service life of thermoset polymers. However, most of the yielded dynamic covalent bonds require an extra catalyst, high temperature and high-pressure conditions to trigger their self-healing properties. Herein, we report on a catalyst-free bis-dynamic covalent polymer network containing vinylogous urethane and disulfide bonds. It is revealed that the introduction of disulfide bonds significantly reduces the activation energy (reduced from 94 kJ/mol to 51 kJ/mol) of the polymer system for exchanging and promotes the self-healing efficiency (with a high efficiency of 86.92% after being heated at 100 °C for 20 h) of the material. More importantly, the mechanical properties of the healed materials are comparable to those of the initial ones due to the special bis-dynamic covalent polymer network. These results suggest that the bis-dynamic covalent polymer network made of disulfide and inter-vinyl ester bonds opens a new strategy for developing high-performance vitrimer polymers.

## 1. Introduction

Traditional thermosets typically exhibit excellent mechanical properties, robust chemical resistance and credible structural stability because of the three-dimensional (3D) permanently cross-linked network structure, and they are widely applied in many fields, such as insulation and packaging materials, textiles, diaphragms and sealants [1]. However, the 3D cross-linked network formed in thermoset polymers renders them infusible and insoluble, hindering their repair and recovery when damaged; thus, the polymers exhibit low reprocessability and recyclability, resulting in significant waste [2]. To overcome this disadvantage, minimize waste and extend the service life of thermoset polymers, a new material that can be repaired while maintaining the properties imparted by the cross-linked network structure is urgently required.

Introducing dynamic covalent bonding into thermoset polymers can be a promising strategy to address these challenges. The dynamic covalent bonding, where the covalent bonds can be reversibly broken–reconfigured in response to external stimuli (e.g., light [3], heat [4,5], pH [6] and chemical stimuli [7]), rearranges the formed cross-linked network, making the network reproducible. In 2011, Leibler et al. [8] first introduced dynamic epoxy–ester bonds into the cross-linked polymer network and demonstrated the recyclability of the thermoset polymers through multiple heat treatments, terming it as a vitrimer. Thereafter, a variety of dynamic covalent bonds were introduced into cross-linked networks [9], including allyl sulfide–thiol exchange [10], boronic ester and oxime ester [11,12], transesterification of (thio) ester [13], transesterification of silyl ether [14], transalkylation of sulfonium salt [15], metathesis of dioxaborolane [16], and the imine and transamination of guanidine [17], etc. Conversely, to guarantee the activity of the transfer reactions of the dynamic chemical bond, catalysts are typically required in the system; however, this poses problems of ageing, spillage and phase separation, which significantly restricts the application of thermosetting polymers. In this regard, to avoid the problems associated with using catalysts, catalyst-free dynamic network systems have been recently reported, such as vinylogous (or enamine-one) urethane [18,19], the transesterification of phosphate triester [20], disulfide exchange [21] and Schiff base covalent systems [22].

Among these dynamic covalent bonds, the vinylogous urethane bond has remarkable advantages because it can be formed by the catalyst-free reaction of primary amines and acetoacetates, and acetoacetate-functional monomers are easily modifiable [23,24,25,26,27,28]. In 2015, Du Prez et al. [29] reported the first vinylogous urethane vitrimer, which was produced using cyclohexanedimethanol diacetoacetate, m-phenylenedimethylamine and tris (2-aminoethyl) amine, and it showed excellent storage modulus (2.4 GPa, at room temperature (RT)), as well as a high capacity for recycling (10 cycles, at 150 °C). Sumerlin et al. [30] produced ethylene vitrimers with acetoacetate-functional groups using cheap commercial monomers; the obtainable vitrimers showed considerable reprocessing ability (4 cycles, at 160 °C) and good mechanical properties (1.8 GPa, at RT). Our group recently synthesized bio-based multifunctional acetoacetate-functional vitrimer from limonene and castor oil, and it demonstrated excellent self-healing properties and can be recycled numerous times [31,32,33]. The vinylogous urethane bond-forming network provides excellent mechanical capabilities and good recyclability. However, this type of network has limited self-healing properties under high-pressure conditions which are crucially important for the applications. 

Recently, we have been interested in the self-healing properties of the dynamic disulfide bonds because of their fast exchange mechanism under mild conditions and their promising results [34,35,36,37]. For example, Kluperman et al. [38] reported self-reparable disulfide bonds containing elastomers from commercial liquid polysulfide resin. The elastomers displayed good mechanical characteristics and can complete self-healing at 60 °C within 1 h. Zhang et al. [39] presented a vitrimer that efficiently facilitates the reversible exchange reaction of aliphatic disulfide bonds under an alkaline condition using tri-n-butylphosphine (TBP). This epoxy matrix exhibited significant mechanical qualities and effective self-healing abilities at RT in 24 h. Thus, the simultaneous introduction of both of the dynamic bonds into the polymer network, with the associated structural adjustments and validation, can offer an excellent solution for developing advanced self-healing materials.

In this study, we developed an acetoacetate monomer via the thiol-click reaction and prepared a bis-dynamic covalent polymer network containing vinylogous urethane and disulfide bonds without a catalyst. Meanwhile, we compared the self-healing performance of the polymer networks without the disulfide bond. After a systematic study, it was revealed that the introduction of the disulfide bonds significantly reduced the activation energy of the polymer system for exchanging and promoted the self-healing efficiency of the material.

## 2. Experimental Part

### 2.1. Experimental Materials

Trimethylolpropane tris (3-mercaptopropionate) (TMPTA), allyl acetoacetate, pentaerythritol tetra (3-mercaptopropionate) (PETMP), and chloroform-d were purchased from Saan Chemical Technology Co., Ltd., Shanghai, China. The 1,6-Diaminohexane (HMDA), cystamine dihydrochloride and 2-hydroxy-2-methylpropiophenone (UV1173) were purchased from Zhiyuan Chemical Reagent Co., Ltd., Tianjin, China. Potassium hydroxide, magnesium sulfate, acetone, and dichloromethane were supplied by Guanghua Sci-Tech Co., Ltd., Shantou, China. All of the chemical materials were used without further purification.

### 2.2. Synthesis of Monomer Trimethylolpropane Tris(3-Mercaptacetoacetate) (TMPTAA) and Pentaerythritol Tetra(3-Mercaptacetoacetate) (PETMPA)

The synthesis of monomer TMPTAA and PETMPA was performed as follows: TMPTA (or PETMP), UV1173 (5 wt.%), and acetone (30 mL) were added to a flask wrapped in tin foil and stirred under nitrogen protection for 10 min. Afterwards, allyl acetoacetate was added into the mixture under a nitrogen atmosphere and stirred for 5 min. Thereafter, the flask was irradiated under an ultraviolet (UV) lamp (8 mW, 365 nm) for 8 h. At the end of the reaction, the solvent was removed and a yellow oil liquid was obtained. 

The yield of TMPTAA was 96% (in Figure S2). ^1^H nuclear magnetic resonance (NMR) (CDCl_3_, 400 MHz): *δ* (ppm) = 4.24 (O-**CH_2_**-CH_2_, t, 6H), 4.06 (O-**CH_2_**-C, s, 6H), 3.49 (O=C-**CH_2_**-C=O, s, 6H), 2.77 (CH_2_-**CH_2_**-CH_2_, t, 6H), 2.64 (**CH_2_**-S-**CH_2_**, q, 6H), 2.27 (**CH_3_**-C=O, s, 8H), 1.94 (CH_2_-**CH_2_**-C=O, m, 6H), 1.52 (C-**CH_2_**-CH_3_, q, 2H), 0.90 (C-**CH_2_**-CH_3_, t, 3H). The ^13^C NMR (CDCl_3_, 100 MHz): *δ* (ppm) = 200.27, 171.26, 166.60, 63.81, 63.48, 49.65, 40.55, 34.48, 29.82, 28.12, 26.71, 22.90, 7.09 (details of NMR are shown in Figures S4 and S5).

The yield of PETMPA was 93% (in Figure S3). ^1^H NMR (CDCl_3_, 400 MHz): *δ* (ppm) = 4.24 (O-**CH_2_**-CH_2_, t, 8H), 4.17 (O-**CH_2_**-C, s, 8H), 3.49 (O=C-**CH_2_**-C=O, s, 8H), 2.76 (CH_2_-**CH_2_**-CH_2_, t, 8H), 2.62 (**CH_2_**-S-**CH_2_**, m, 16H), 2.28 (**CH_3_**-C=O, s, 12H), 1.94 (CH_2_-**CH_2_**-C=O, m, 8H). The ^13^C NMR (CDCl_3_, 100 MHz): *δ* (ppm) = 200.48, 171.05, 167.03, 63.70, 62.21, 49.86, 42.10, 34.34, 30.10, 28.27, 26.78 (details of NMR are shown in Figures S6 and S7).

### 2.3. Synthesis of Cystamine (Cys)

Cystamine dihydrochloride (8.00 g, 35.5 mmol) and water (80 mL) were added to a 250-mL beaker. After dissolving, potassium hydroxide (KOH) (6.00 g, 107.0 mmol) was added, and the mixture was stirred for 1 h. The resulting mixture was extracted with 4 × 150 mL of dichloromethane; the collected organic layer was passed through anhydrous MgSO_4_ and dried via spin evaporation at 45 °C to afford 4.3 g of light-yellow oil (yield 80%).

### 2.4. Synthesis of Polymer Films

**Scheme 1:**
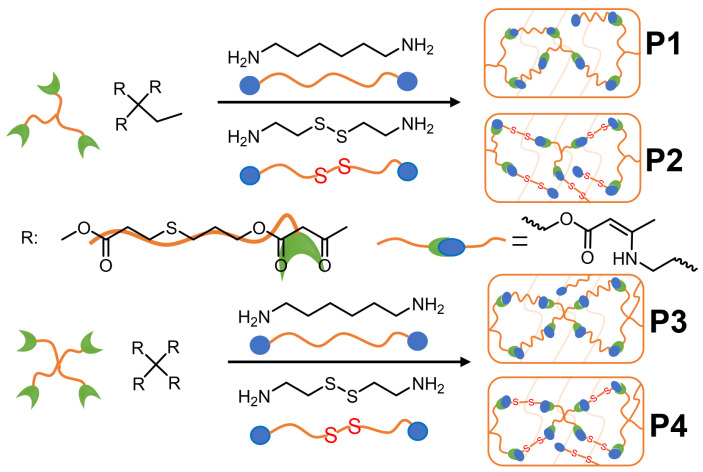
Synthesis of the polymer films.

The synthesis route and composition of the polymer films are shown in Scheme 1 and Table 1, and the experimental procedure is as follows: TMPTAA (or PETMPA), amine curing agent and acetone (6 mL) were added to a 10-mL weighing bottle and stirred for 5 min. Afterwards, the mixture was poured into a polytetrafluoroethylene (PTFE) mold (8 × 8 × 2 cm) and cured at 30 °C for 12 h. Finally, the polymer films with a thickness of 500 μm were obtained.

### 2.5. Characterization

***Nuclear Magnetic Resonance (NMR) Spectroscopy.*** ^1^H NMR (400 MHz) and ^13^C NMR (100 MHz) spectra were recorded by Bruker AV-400 spectrometer. Chemical shift (δ) was expressed in parts per million (ppm), and each sample was tested with CDCl_3_ as a solvent.

***FT-IR Spectroscopy.*** The structure of prepolymers and polymers were characterized on a Nicolet iS20 FT-IR spectrometer from 4000 cm^−1^ to 500 cm^−1^ with setting resolution to 0.25 cm^−1^, each sample was scanned 32 times using a diamond ATR probe.

***Thermal Gravimetric Analysis (TGA).*** Thermogravimetric analysis (TGA) experiments were carried out on a TA-Q50 thermogravimetric analyzer, ramped up from 25 °C to 750 °C at a rate of 10 °C/min in a nitrogen atmosphere. 

***Differential Scanning Calorimetry (DSC)*.** DSC experiments were performed using DSC-Q2000 under a nitrogen atmosphere from −40 °C to 120 °C at a rate of 20 °C/min. 

***Dynamic Mechanical Analysis (DMA)***. Dynamic Mechanical Analysis (DMA) was conducted with a TA Q800 (New Castle, DE, USA) instrument. Each sample had a size of a 7 mm × 3 mm × 0.5 mm rectangular shape. For the DMA experiments, a preloaded axial force of 0.01 N, a displacement amplitude of 10 μm, and a regular frequency of 1 Hz were preloaded. Each sample was heated to 180 °C at −3 °C/min at −20 °C. The tensile test was performed using DMAQ800 and the applied force was increased with a rate of 3 N/min to 18 N. Stress Relaxation Analysis (SRA) experiments were conducted at the setting strain of 1% and a temperature range of 60 °C–130 °C. All of the DMA experiments results were collected through TA’s Thermal Advantage for Q Series software New Castle, DE, USA. 

***Self-Healing Experiments.*** The film (long × wide × high= 7 mm × 3 mm × 0.5 mm) was cut in half using a knife, put into contact without further pressure and placed in a 100 °C environment for self-healing experiments. The optical micrographs of the self-healed samples were obtained during the healing process using a digital camera attached to a Meiji optical microscope. The tensile test was performed using the DMAQ800, the self-healing efficiency was measured and were as detailed in the previous section. All measurements were averaged by at least four measurements. The self-healing efficiency (*η*) of the polymer was determined by the ratio of the tensile strength of the self-healed sample ($\sigma_{\mathrm{healed}}$) to that of the initial sample ($\sigma_{\mathrm{initial}}$), and the self-healing efficiency (*η*) was calculated as follows [31]:

$$\eta =\frac{\sigma_{\mathrm{healed}}}{\sigma_{\mathrm{initial}}}$$

***Swelling experiments.*** The swelling ratio (SR) of the polymer films was detected as follows. A dried polymer film (2 × 1 cm^2^) was first weighed and recorded as W_1_ and immersed in an Ace solvent for several hours. The weight of the wet polymer film was recorded as W_2_ after quickly wiping its surface with a filter paper. The SR of the films in the Ace solvent was calculated as follows: M (%) = [(W_2_ − W_1_)/W_1_] × 100%. The gel content (GC) was determined by cutting a polymer film of mass W_1_, soaking it in the Ace solvent for 48 h, and drying it at 80 °C for 48 h; the sample weight was denoted as W_3_. The GC was obtained with the following equation: M (%) = W_3_/W_1_ × 100%.

The cross-link density ($v_{e}$) of the polymer film was calculated using the Flory–Rhener relationship [40,41,42,43]:

$$v_{e}=\frac{-\left[\ln\left(1-v_{2}\right)+v_{2}+\chi v_{2}{}^{2}\right]}{V_{s}\left(v_{2}{}^{1/3}-v_{2}/2\right)},$$

where $v_{s}$ is the molar volume of the solvent; $v_{2}$ is the volume fraction of the swollen polymer; and χ is the polymer–solvent interaction parameter.

The volume fraction ($v_{2}$) of the dissolved polymer was calculated according to the following formula:

$$v_{2}=\frac{W_{3}/\rho_{\mathrm{polymer}}}{W_{3}/\rho_{\mathrm{polymer}}+W_{2}/\rho_{\mathrm{acetone}}},$$

where $\rho_{\mathrm{acetone}}$ is 0.788 g/cm; and $\rho_{\mathrm{polymer}}$ is the density of the polymer.

The polymer–solvent interaction parameter χ was calculated as follows:

$$\chi =0.34+\frac{V_{s}}{\mathrm{RT}}{\left(\delta_{\mathrm{polymer}}-\delta_{\mathrm{acetone}}\right)}^{2},$$

where R is the gas constant (8.314 J/K/mol); and T is the absolute temperature (273 K). Additionally, for acetone, $v_{s}$ is 73 cm^3^/mol, $\delta_{\mathrm{polymer}}$ is the solubility parameter of the polymer, $\delta_{\mathrm{acetone}}$ is the solubility parameter of acetone and $\delta_{\mathrm{acetone}}$ is 20.4 (J/cm^3^)^0.5^, while $\delta_{\mathrm{polymer}}$ can be determined by the solubility method.

## 3. Results and Discussion

### 3.1. Synthesis of Acetoacetic Acid Esterified Functional Monomers (TMPTAA and PETMPA)

**Figure 1:**
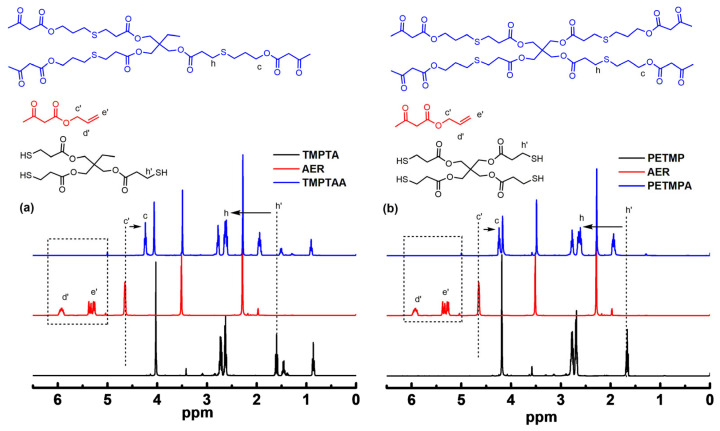
(**a**) ^1^H NMR of TMPTA, PETMP, allyl acetoacetate and TMPTAA, (**b**) ^1^H NMR of TMPTA, PETMP, allyl acetoacetate and PETMPA.

TMPTAA was synthesized from TMPTA and allyl acetoacetate by the thiol-click reaction under 365 nm UV light. This preparation method is a highly efficient, direct and fast method to obtain the functional monomers of acetoacetate without by-products. Figure 1 shows the ^1^H NMR spectra of the monomers. For TMPTAA (Figure 1a), the –CH_2_– proton peak h’ next to the thiol group of TMPTA shifted from 1.59 to 2.62 ppm (proton peak h, –CH_2_– group adjacent to the thioether), and the –CH_2_– proton peak c’ of AER shifted from 4.63 to 4.23 ppm (proton peak c, –CH_2_– group adjacent to the ether) after the thiol-click reaction. Combined with the disappearance of the proton peaks e’ and d’ assigned to the double bond, the TMPTAA was successfully prepared. The same chemical shift change is observed in Figure 1b, implying that the monomer PETMPA was obtained.

**Figure 2:**
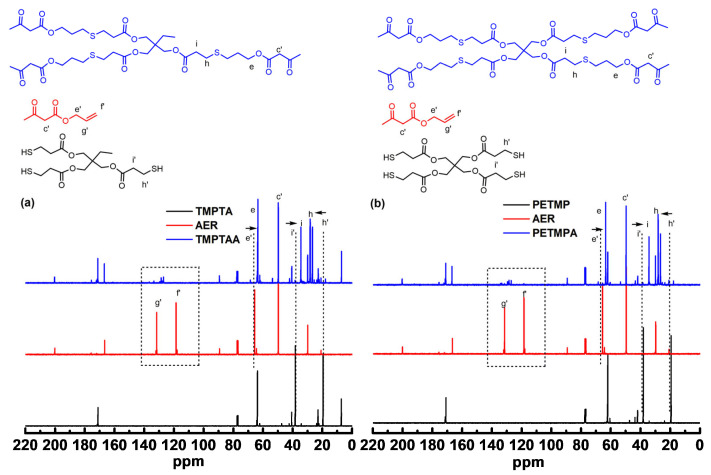
(**a**) ^13^C NMR of TMPTA, AER and TMPTAA, (**b**) ^13^C NMR of TMPTA, AER and PETMPA.

Further, the ^13^C NMR spectra of TMPTAA and PETMPA is shown in Figure 2. After the thiol-click reaction between AER and TMPTA, the peak position changed. Specifically, the C=C bond signal peaks g’ and f’ of AER at 131 ppm and 181 ppm disappeared, while the methylene signal peak e’ adjacent to the AER ester group at 65 ppm shifted to 63 ppm. Further, the methylene signal peak h’ next to the TMPTA thiol group at 19 ppm shifted to 28 ppm, and the methylene signal peak i’ near the TMPTA carbonyl group at 38 ppm shifted to 35 ppm. Similar changes in the NMR signal peaks occurred in PETMPA. These results showed that the thiol-click reaction was performed, and the acetoacetate-functionalized TMPTAA and PETMPA were prepared.

### 3.2. Synthesis and Characterization of the Polymer Films

**Figure 3:**
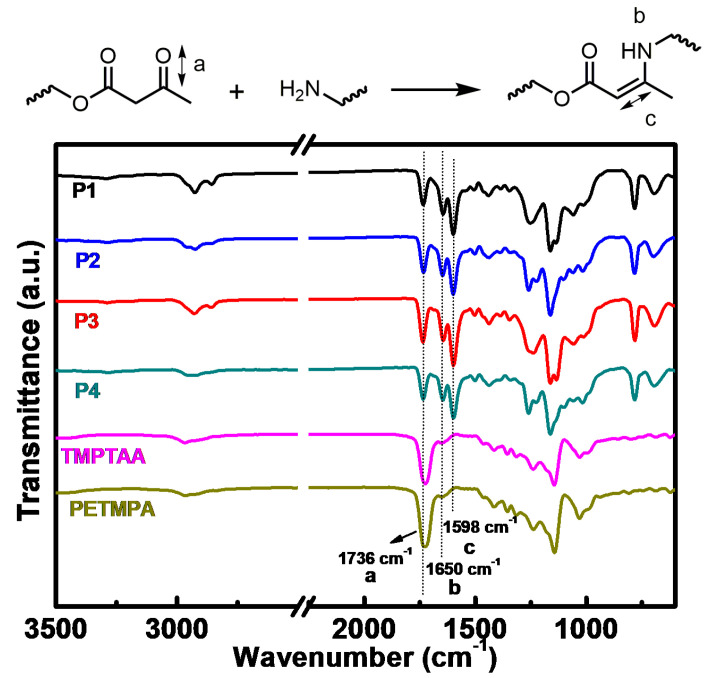
FT-IR spectra of TMPTAA, PETMPA and polymer films.

The chemical structure of the TMPTAA, PETMPA and four polymer networks were characterized by FT-IR and are shown in Figure 3. Although all films showed similar characteristic IR absorption peaks, there were changes in the density and position of several absorption peaks, when compared with monomers. Typically, the peak at 1736 cm^−1^ (C=O stretching vibration in TMPTAA and PETMPA) was dramatically weakened, and new absorption peaks appeared at 1650 cm^−1^ (C=C stretching vibration) and 1598 cm^−1^ (C=O stretching vibration) [32,44,45,46]. Further, the detection of the cross-link structure was verified by acetone swelling experiments, where ca. 0.1 g of the polymer network was submerged in acetone, and the swelling rate of the film was measured over time (Figure S1). The films were not dissolved, only swollen, after being immersed for 48 h (see Table 2), demonstrating that the crosslinking films were successfully prepared.

**Figure 4:**
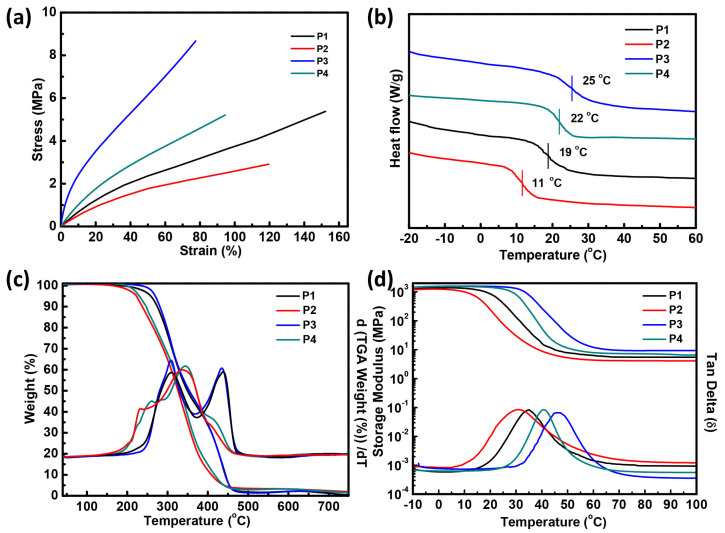
(**a**) Stress–strain curve, (**b**) DSC curve, (**c**) TGA curve and (**d**) DMA curve of the films.

Figure 4a shows the mechanical properties of the polymer films. The mechanical properties gradually increased with the increase in the cross-link density (Figure S1; Table 2 and Table 3). Crosslinking density showed a trend of P3 > P4 > P1 > P2, and the Young’s Modulus from 6.0 MPa (P2) to 31.3 MPa (P3). All of the tensile data are recorded in Table 3 and it was observed that the Young’s Modulus increased as the crosslinking density increased with more acetoacetate-functional groups in the crosslinking substrate (P3 > P1 and P4 > P2). Moreover, the films prepared from 1,6-hexanediamine posed better Young’s Modulus than the cystamine (P1 > P2 and P3 > P4). Figure 4b shows the DSC curves of the films; the *T*_g_ of the films were in the order of P3 (25 °C) > P4 (22 °C) > P1 (19 °C) > P2 (11 °C). Note that the films prepared with cystamine had a lower *T*_g_ than those made with 1,6-hexanediamine, while those synthesized with trifunctional TMPTA had a lower *T*_g_ than those with PETMPA. The increased cross-link density of the polymer films resulted in a corresponding increase in *T*_g_. The TGA curves showed the thermal stability of the films, as shown in Figure 4c, where the cystamine-prepared films, P2 and P4, exhibited a 10% weight loss at 210 °C, while the 1,6-hexanediamine-prepared films, P1 and P3, exhibited 10% weight loss at 250 °C. The relatively low thermal-induced weight loss temperature of the P2 and P4 films was attributed to the unstable breakage of the disulfide bond at 210 °C.

The dynamic mechanical properties of the films were investigated by DMA experiments. As shown in Figure 4d, all of the samples showed an obvious rubber platform after the glass transition. We calculated the crosslinking density of each film by using the cross-linking density calculation formula: *v*_e_ = *E*’/3*RT* [47], where *E*’ represents the energy storage modulus under *T*_g_ + 50 °C, *R* refers to the gas constant, and *T* is *T*_g_ + 50 °C. *T*_g_ is closely correlated with the cross-linking density and transferred from 30 °C to 46 °C, with *T*_g_ increasing with the number of prepolymer acetoacetate groups, and lower cystamine-prepared films compared to 1,6-diaminohexane-prepared films, which is consistent with the results obtained by DSC (with the same trend as *T*_g_). This suggests that the mechanical properties of the film can be effectively adjusted according to the amount of regulated prepolymer acetoacetate groups of flexible chain-segment disulfide monomers. In addition, the addition of cystamine reduces the mechanical properties of the film.

### 3.3. Stress-Relaxation Experiments

**Figure 5:**
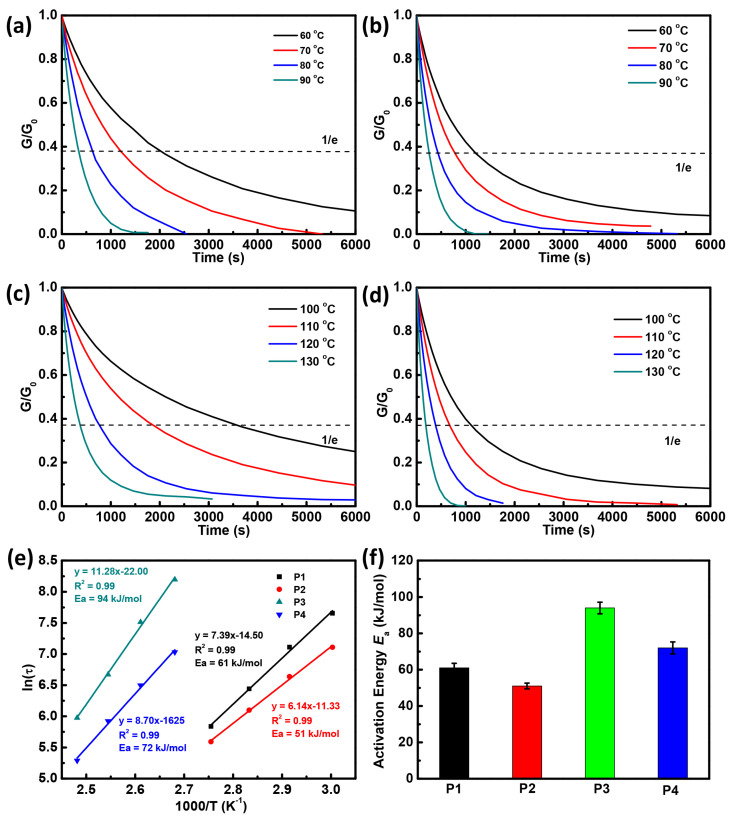
Normalized stress-relaxation curves for (**a**) films P1, (**b**) P2, (**c**) P3 and (**d**) P4 at different temperatures; (**e**) the Arrhenius analysis of P1, P2, P3 and P4; (**f**) the histogram of the activation energy of the films.

Stress-relaxation measurement was taken to investigate the viscoelasticity of the polymer films and the rate of exchange reactions within the material. The relaxation time *τ* is defined as the time taken for the material to relax from the initial modulus to 1/e of the initial modulus. Figure 5a–d shows the stress-relaxation curves for the polymer films. The relaxation time for P1 decreased from 1997 s to 343 s as the test temperature increased from 60 °C to 90 °C (Figure 5a), while the relaxation time for P2 decreased from 1211 s to 250 s (Figure 5b). Additionally, as the test temperature increased from 100 °C to 130 °C, the relaxation time decreased from 3487 s to 373 s for P3 (Figure 5c), and from 1110 s to 174 s for P4 (Figure 5d). To deeply study the energy required for the macroscopic flow of the material, the activation energy (*E*_a_) of polymer films was calculated using the Arrhenius equation: *τ* = *τ*_0_ × exp (*E*_a_/*RT*) [48,49,50,51] and is shown in Figure 5c,d. The *E*a of the polymer films was in the following order: P3 (94 kJ/mol) > P4 (72 kJ/mol) > P1 (61 kJ/mol) > P2 (51 kJ/mol), demonstrating that the disulfide bond decreased the activation energy for the same type of film system.

### 3.4. Self-Healing Property of the Polymer Films

**Figure 6:**
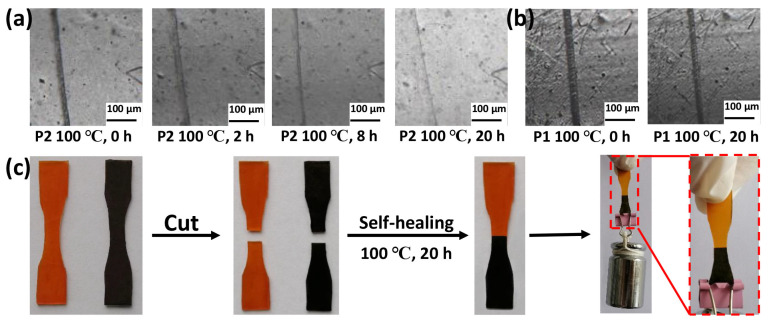
(**a**) Photographs of scratch self-healed P2 at 100 °C for 0 h, 2 h, 8 h and 20 h; (**b**) the photographs of scratch self-healed P1 at 100 °C for 0 h and 20 h; (**c**) dumbbell-shaped film P2 coated with different colors was spliced, and it self-healed at 100 °C for 20 h, and the self-healed film was suspended from a 500 g weight.

To determine the effect of the disulfide in the film system on the self-healing properties of the films, the strip samples were cut and heated in a 100 °C oven for 2 h, 8 h and 20 h. Thereafter, their morphological changes during the repair process were visualized using an optical microscope to ascertain the variation in the self-healing abilities over time. Figure 6a,b, depict the photographs of healed P2 and P1, respectively. The scratch on the surface of P2 was visible when it was first cut off, and became smaller as the healing time increased. When the healing time reached 20 h, most scratches on the P2 surface disappeared, and healed P2 could suspend a 500 g weight (Figure 6c). However, no significant scratch healing was observed after P1 was treated at 100 °C for 20 h. These results demonstrated that the introduction of the disulfide bond results in the excellent self-healing ability of the films. 

**Figure 7:**
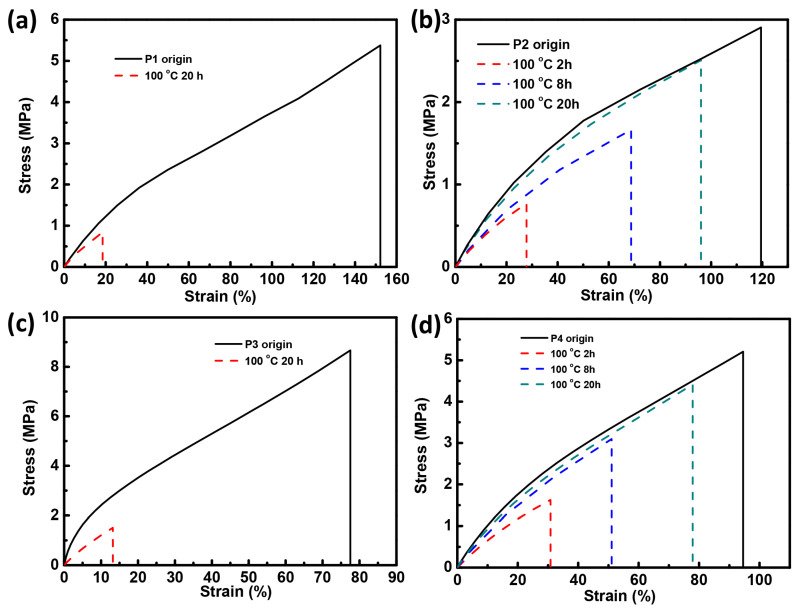
Stress–strain test of the self-healing efficiency of the films: (**a**) film P1 and (**c**) film P3 with inefficient stress–strain recovery after 20 h of healing at 100 °C, and (**b**) film P2 and (**d**) film P4 with basic recovered stress–strain after 20 h of self-healing at 100 °C.

To further investigate the self-healing properties and healing mechanisms of the polymer films, their self-healing efficiency was quantified by stress–strain experiments. Four film samples were cut and healed by heating in an oven at 100 °C. Figure 7 and Table 4 show the self-healing efficiency of P1, P2, P3 and P4 healed at 100 °C at different times. A significant difference was observed between the self-healing efficiency of the films containing the disulfide bonds and those without the disulfide bonds at different healing times. With the extension of the healing time from 2 h to 20 h, the molecular chains diffused into the damaged area more deeply, facilitating the reorganization of dissociated disulfide and hydrogen bonds [21]. In particular, the P4 film healed at 100 °C for 20 h with a high self-healing efficiency of 86.92%, which was 50.57% higher than that for 2 h. Moreover, P4 showed the highest stress recovery in the same healing time, although the difference in the self-healing efficiency between P2 and P4 was only 0.67% (20 h healing). As shown in Figure 7a–d, the self-healing efficiency of P4 and P2 showed an increasing trend, while P1 and P3 were not significantly healed for 20 h. This indicates that the introduction of the disulfide bonds enhanced the movement of the elastomer molecular chain segments and increased the self-healing efficiency [43,52].

## 4. Conclusions

In conclusion, a series of novel polymer films containing vinylogous urethane and disulfide bonds were developed from multifunctional acetoacetate (TEMPTAA and PETMPA) and modified Cys. The experimental results indicated that the high functional group equipped with acetoacetate is good for improving the crosslinking density of the polymer network. The stress-relaxation results showed that the Cys films prepared from the same functional monomer yielded relatively low activation energy and high chain segment movement because of the low crosslinking density resulting in low *T*_g_ and Young’s modulus. The equipped low-activation energy (e.g., P2: *E*_a_ = 51 kJ/mol) ensured a fast exchange of the dynamic disulfide bonds. Thus, the disulfide-containing films presented excellent self-healing ability, with a repair efficiency of 86.92% for P4 heated at 100 °C for 20 h. Additionally, all of the polymer films containing vinylogous urethane double bonds exhibited excellent mechanical properties. It can be concluded that the strategy of introducing dynamic disulfide and vinylogous urethane bonds can provide excellent mechanical properties and self-healing abilities to the materials, which will provide a potential possibility for future research on vitrimer polymers. 

## Figures and Tables

**Table 1:** Materials for the polymer film preparation.

Sample	Acetoacetate	Primary Amine	Acetoacetate/Amine Ratio
P1	TMPTAA (0.83 g, 1.00 mmol)	1,6-Didiamine (0.18 g, 1.58 mmol)	1:1.05
P2	TMPTAA (0.83 g, 1.00 mmol)	Cystamine (0.24 g, 1.58 mmol)	1:1.05
P3	PETMPA (1.05 g, 1.00 mmol)	1,6-Didiamine (0.24 g, 2.1 mmol)	1:1.05
P4	PETMPA (1.05 g, 1.00 mmol)	Cystamine (0.32 g, 2.1 mmol)	1:1.05

**Table 2:** Film swelling data.

Sample	Gel Content (%)	Swelling Ratio (%)	*v*_e_ (mol cm^−3^)
P1	94	42	1.24 × 10^−4^
P2	91	48	0.83 × 10^−4^
P3	95	32	5.86 × 10^−4^
P4	92	36	2.79 × 10^−4^

**Table 3:** Mechanical and thermal properties of the films.

Sample	Young’s Modulus (MPa)	Stress at Break (MPa)	Elongation at Breaks (%)	*T*_g_ (DSC) (°C)	TGA in Nitrogen (°C)			*T*_g_ (tan*δ*) (°C)	*E’* at *T*_g_ + 50 °C (MPa)	Crosslinking *(v_e_)* (mol m^−3^*)*
					T_10_	T_50_	T_max_			
P1	7.3 ± 0.3	5.37 ± 0.46	152.1 ± 10.2	19	274	349	474	34	5.52	619
P2	6.0 ± 0.5	2.91 ± 0.32	119.5 ± 5.3	11	237	326	465	30	4.17	473
P3	31.3 ± 1.2	8.67 ± 1.13	77.5 ± 6.5	22	280	354	480	46	9.41	1022
P4	10.8 ± 0.7	5.20 ± 0.43	94.5 ± 7.6	25	247	332	468	41	6.84	753

**Table 4:** Film self-healing efficiency.

Sample	Healing for Time	The Temperature of Self-Healing (°C)	Stress at Break σ (MPa)	Self-Healing Efficiency *η* (%)
P1	before cutting	-	5.37	-
	20 h	100	0.84	15.64
P2	before cutting	-	2.91	-
	2 h	100	0.76	26.12
	8 h	100	1.66	57.04
	20 h	100	2.51	86.25
P3	before cutting	-	8.67	-
	20 h	100	1.51	17.41
P4	before cutting	-	5.20	-
	2 h	100	1.63	31.35
	8 h	100	3.09	59.4
	20 h	100	4.52	86.92

## Data Availability

All data generated or analyzed during this study are included in the published articles included in this article.

## Supplementary Materials

The following supporting information can be downloaded at: https://www.mdpi.com/article/10.3390/polym14193953/s1, Figure S1: Solvent swelling (acetone absorption-time diagram) of P1, P2, P3 and P4; Figure S2: ^1^H NMR Spectrum of (a) allyl acetoacetate and (b) trimethylolpropane tris(3-mercaptacetoacetate) (TMPTAA) (400 MHz, CDCl_3_, 7.26 ppm); Figure S3: ^1^H NMR Spectrum of (a) allyl acetoacetate and (b) pentaerythritol tetra(3-mercaptacetoacetate) (PETMPA) (400 MHz, CDCl_3_, 7.26 ppm); Figure S4: ^1^H NMR Spectrum of trimethylolpropane tris(3-mercaptacetoacetate) (TMPTAA) (400 MHz, CDCl_3_, 7.26 ppm); Figure S5: ^13^C NMR Spectrum of trimethylolpropane tris(3-mercaptacetoacetate) (TMPTAA) (100 MHz, CDCl_3_, 77 ppm); Figure S6: ^1^H NMR Spectrum of pentaerythritol tetra(3-mercaptacetoacetate) (PETMPA) (400 MHz, CDCl_3_, 7.26 ppm); Figure S7: ^13^C NMR Spectrum of pentaerythritol tetra(3-mercaptacetoacetate) (PETMPA) (100 MHz, CDCl_3_, 77 ppm).

## References

[1] Raquez J.M., Deléglise M., Lacrampe M.F., Krawczak P. (2010). Thermosetting (bio)materials derived from renewable resources: A critical review. Prog. Polym. Sci..

[2] Krishnakumar B., Pucci A., Wadgaonkar P.P., Kumar I., Binder W.H., Rana S. (2022). Vitrimers based on bio-derived chemicals: Overview and future prospects. Chem. Eng. J..

[3] Zhao P., Xia J., Cao M., Xu H. (2020). Wavelength-Controlled Light-Responsive Polymer Vesicle Based on Se–S Dynamic Chemistry. ACS Macro Lett..

[4] Aiba M., Koizumi T.-a., Futamura M., Okamoto K., Yamanaka M., Ishigaki Y., Oda M., Ooka C., Tsuruoka A., Takahashi A. (2020). Use of Bis(2,2,6,6-tetramethylpiperidin-1-yl)trisulfide as a Dynamic Covalent Bond for Thermally Healable Cross-Linked Polymer Networks. ACS Appl. Polym. Mater..

[5] Tang Z., Lyu X., Xiao A., Shen Z., Fan X. (2018). High-Performance Double-Network Ion Gels with Fast Thermal Healing Capability via Dynamic Covalent Bonds. Chem. Mater..

[6] Wang Y., Xing P., Li S., Ma M., Yang M., Zhang Y., Wang B., Hao A. (2016). Facile Stimuli-Responsive Transformation of Vesicle to Nanofiber to Supramolecular Gel via ω-Amino Acid-Based Dynamic Covalent Chemistry. Langmuir.

[7] Kiran B.M., Jayaraman N. (2009). Thiol−Disulfide Interchange Mediated Reversible Dendritic Megamer Formation and Dissociation. Macromolecules.

[8] Montarnal D., Capelot M., Tournilhac F., Leibler L. (2011). Silica-Like Malleable Materials from Permanent Organic Networks. Science.

[9] Cardenas C.B., Gayraud V., Rodriguez M.E., Costa J., Salaberria A.M., de Luzuriaga A.R., Markaide N., Keeryadath P.D., Zapateria D.C. (2022). Study into the Mechanical Properties of a New Aeronautic-Grade Epoxy-Based Carbon-Fiber-Reinforced Vitrimer. Polymers.

[10] Worrell B.T., McBride M.K., Lyon G.B., Cox L.M., Wang C., Mavila S., Lim C.-H., Coley H.M., Musgrave C.B., Ding Y. (2018). Bistable and photoswitchable states of matter. Nat. Commun..

[11] Zhang X., Wang S., Jiang Z., Li Y., Jing X. (2020). Boronic Ester Based Vitrimers with Enhanced Stability via Internal Boron–Nitrogen Coordination. J. Am. Chem. Soc..

[12] He C., Shi S., Wang D., Helms B.A., Russell T.P. (2019). Poly(oxime–ester) Vitrimers with Catalyst-Free Bond Exchange. J. Am. Chem. Soc..

[13] Shi Q., Yu K., Kuang X., Mu X., Dunn C.K., Dunn M.L., Wang T., Qi H.J. (2017). Recyclable 3D printing of vitrimer epoxy. Mater. Horiz..

[14] Nishimura Y., Chung J., Muradyan H., Guan Z. (2017). Silyl Ether as a Robust and Thermally Stable Dynamic Covalent Motif for Malleable Polymer Design. J. Am. Chem. Soc..

[15] Hendriks B., Waelkens J., Winne J.M., Du Prez F.E. (2017). Poly(thioether) Vitrimers via Transalkylation of Trialkylsulfonium Salts. ACS Macro Lett..

[16] Wu S., Yang H., Huang S., Chen Q. (2020). Relationship between Reaction Kinetics and Chain Dynamics of Vitrimers Based on Dioxaborolane Metathesis. Macromolecules.

[17] Garcia J.M., Jones G.O., Virwani K., McCloskey B.D., Boday D.J., ter Huurne G.M., Horn H.W., Coady D.J., Bintaleb A.M., Alabdulrahman A.M.S. (2014). Recyclable, strong thermosets and organogels via paraformaldehyde condensation with diamines. Science.

[18] Chen Y., Zhang H., Majumdar S., van Benthem R.A.T.M., Heuts J.P.A., Sijbesma R.P. (2021). Dynamic Polyamide Networks via Amide–Imide Exchange. Macromolecules.

[19] Guo X., Gao F., Chen F., Zhong J., Shen L., Lin C., Lin Y. (2021). Dynamic Enamine-one Bond Based Vitrimer via Amino-yne Click Reaction. ACS Macro Lett..

[20] Majumdar S., Zhang H., Soleimani M., van Benthem R.A.T.M., Heuts J.P.A., Sijbesma R.P. (2020). Phosphate Triester Dynamic Covalent Networks. ACS Macro Lett..

[21] Zhang C., Liang H., Liang D., Lin Z., Chen Q., Feng P., Wang Q. (2021). Renewable Castor-Oil-based Waterborne Polyurethane Networks: Simultaneously Showing High Strength, Self-Healing, Processability and Tunable Multishape Memory. Angew. Chem. Int. Ed..

[22] Wang S., Ma S., Li Q., Xu X., Wang B., Yuan W., Zhou S., You S., Zhu J. (2019). Facile in situ preparation of high-performance epoxy vitrimer from renewable resources and its application in nondestructive recyclable carbon fiber composite. Green Chem..

[23] Taplan C., Guerre M., Winne J.M., Du Prez F.E. (2020). Fast processing of highly crosslinked, low-viscosity vitrimers. Mater. Horiz..

[24] Liu Z., Zhang C., Shi Z., Yin J., Tian M. (2018). Tailoring vinylogous urethane chemistry for the cross-linked polybutadiene: Wide freedom design, multiple recycling methods, good shape memory behavior. Polymer.

[25] Spiesschaert Y., Guerre M., Imbernon L., Winne J.M., Du Prez F. (2019). Filler reinforced polydimethylsiloxane-based vitrimers. Polymer.

[26] Lessard J.J., Scheutz G.M., Sung S.H., Lantz K.A., Epps T.H., Sumerlin B.S. (2019). Block copolymer vitrimers. J. Am. Chem. Soc..

[27] Guerre M., Taplan C., Nicolaÿ R., Winne J.M., Du Prez F.E. (2018). Fluorinated vitrimer elastomers with a dual temperature response. J. Am. Chem. Soc..

[28] Tellers J., Pinalli R., Soliman M., Vachon J., Dalcanale E. (2019). Reprocessable vinylogous urethane cross-linked polyethylene via reactive extrusion. Polym. Chem..

[29] Denissen W., Rivero G., Nicolaÿ R., Leibler L., Winne J.M., Du Prez F.E. (2015). Vinylogous urethane vitrimers. Adv. Funct. Mater..

[30] Lessard J.J., Garcia L.F., Easterling C.P., Sims M.B., Bentz K.C., Arencibia S., Savin D.A., Sumerlin B.S. (2019). Catalyst-free vitrimers from vinyl polymers. Macromolecules.

[31] Wei X., Ge J., Gao F., Chen F., Zhang W., Zhong J., Lin C., Shen L. (2021). Bio-based self-healing coating material derived from renewable castor oil and multifunctional alamine. Eur. Polym. J..

[32] Zhu Y., Gao F., Zhong J., Shen L., Lin Y. (2020). Renewable castor oil and DL-limonene derived fully bio-based vinylogous urethane vitrimers. Eur. Polym. J..

[33] Chen F., Cheng Q., Gao F., Zhong J., Shen L., Lin C., Lin Y. (2021). The effect of latent plasticity on the shape recovery of a shape memory vitrimer. Eur. Polym. J..

[34] Wu X., Liu M., Zhong J., Zhong Y., Rong J., Gao F., Qiao Y., Shen L., He H. (2022). Self-healing dynamic bond-based robust polyurethane acrylate hybrid polymers. New J. Chem..

[35] Rong J., Zhong J., Yan W., Liu M., Zhang Y., Qiao Y., Fu C., Gao F., Shen L., He H. (2021). Study on waterborne self-healing polyurethane with dual dynamic units of quadruple hydrogen bonding and disulfide bonds. Polymer.

[36] Li Z.-J., Zhong J., Liu M.-C., Rong J.-C., Yang K., Zhou J.-Y., Shen L., Gao F., He H.-F. (2020). Investigation on self-healing property of epoxy resins based on disulfide dynamic links. Chin. J. Polym. Sci..

[37] Liu M., Zhong J., Li Z., Rong J., Yang K., Zhou J., Shen L., Gao F., Huang X., He H. (2020). A high stiffness and self-healable polyurethane based on disulfide bonds and hydrogen bonding. Eur. Polym. J..

[38] Canadell J., Goossens H., Klumperman B. (2011). Self-healing materials based on disulfide links. Macromolecules.

[39] Lei Z.Q., Xiang H.P., Yuan Y.J., Rong M.Z., Zhang M.Q. (2014). Room-Temperature Self-Healable and Remoldable Cross-linked Polymer Based on the Dynamic Exchange of Disulfide Bonds. Chem. Mater..

[40] Hayashi M., Yano R. (2020). Fair Investigation of Cross-Link Density Effects on the Bond-Exchange Properties for Trans-Esterification-Based Vitrimers with Identical Concentrations of Reactive Groups. Macromolecules.

[41] Xie H., Cheng C.-Y., Deng X.-Y., Fan C.-J., Du L., Yang K.-K., Wang Y.-Z. (2017). Creating Poly (tetramethylene oxide) Glycol-Based Networks with Tunable Two-Way Shape Memory Effects via Temperature-Switched Netpoints. Macromolecules.

[42] Cao Z., Gao F., Zhao J., Lin C., Zhong J., Chen F., Zhu Y., Yi P., Shen L. (2019). Ambient Cross-linking System Based on the Knoevenagel Condensation Reaction between Acetoacetylated Sucrose and Aromatic Dicarboxaldehydes. ACS Omega.

[43] Zhu L., Xu L., Jie S., Li B. (2021). Polybutadiene Vitrimers with Tunable Epoxy Ratios: Preparation and Properties. Polymers.

[44] Zuo H., Cao Z., Shu J., Xu D., Zhong J., Zhao J., Wang T., Chen Y., Gao F., Shen L. (2019). Effect of structure on the properties of ambient-cured coating films prepared via a Michael addition reaction based on an acetoacetate-modified castor oil prepared by thiol-ene coupling. Prog. Org. Coat..

[45] Xu D., Cao Z., Wang T., Zhao J., Zhong J., Xiong P., Wang J., Gao F., Shen L. (2019). Effect of the Ratio of Acetylacetate Groups on the Properties of a Novel Plant-Based Dual-Cure Coating System. ACS Omega.

[46] Xu D., Cao Z., Wang T., Zhong J., Zhao J., Gao F., Luo X., Fang Z., Cao J., Xu S. (2019). An ambient-cured coating film obtained via a Knoevenagel and Michael addition reactions based on modified acetoacetylated castor oil prepared by a thiol-ene coupling reaction. Prog. Org. Coat..

[47] Di Mauro C., Genua A., Mija A. (2021). Kinetical Study, Thermo-Mechanical Characteristics and Recyclability of Epoxidized Camelina Oil Cured with Antagonist Structure (Aliphatic/Aromatic) or Functionality (Acid/Amine) Hardeners. Polymers.

[48] Van Lijsebetten F., De Bruycker K., Winne J.M., Du Prez F.E. (2022). Masked Primary Amines for a Controlled Plastic Flow of Vitrimers. ACS Macro Lett..

[49] Debsharma T., Amfilochiou V., Wróblewska A.A., De Baere I., Van Paepegem W., Du Prez F.E. (2022). Fast Dynamic Siloxane Exchange Mechanism for Reshapable Vitrimer Composites. J. Am. Chem. Soc..

[50] Chen F., Gao F., Zhong J., Shen L., Lin Y. (2020). Fusion of Biobased Vinylogous Urethane Vitrimers with Distinct Mechanical Properties. Mater. Chem. Front..

[51] Chen L., Zhu S., Toendepi I., Jiang Q., Wei Y., Qiu Y., Liu W. (2020). Reprocessable, Reworkable, and Mechanochromic Polyhexahydrotriazine Thermoset with Multiple Stimulus Responsiveness. Polymers.

[52] Fortman D.J., Snyder R.L., Sheppard D.T., Dichtel W.R. (2018). Rapidly Reprocessable Cross-Linked Polyhydroxyurethanes Based on Disulfide Exchange. ACS Macro Lett..

