# Supplementary material for: Self-Healable Covalently Adaptable Networks Based on Disulfide Exchange
Source: Polymers (Basel). 2022 Sep 21;14(19):3953. doi: 10.3390/polym14193953 (PMC9570560; doi:10.3390/polym14193953)
Supplement: Supplementary file 1 [file polymers-14-03953-s001.zip › polymers-1893536-supplementary.pdf]

# Supporting Information

## Self-healable covalently adaptable networks based on disulfide exchange

Xinru Guo[a], Feng Liu[a]*, Meng Lv[a], Fengbiao Chen[a], Fei Gao[a]*, Zhenhua Xiong[a], Xuejiao Chen[a], Xuelang Gao[b]*

[a]Jiangxi Engineering Laboratory of Waterborne Coating, School of Chemistry and Chemical Engineering, Jiangxi Science &Technology Normal University, Nanchang, Jiangxi, 330013, P. R. China.

[b]Department of Chemistry, Pohang University of Science and Technology (POSTECH), Pohang, Korea 790-784

E-mail: feng67807@163.com (F. Liu), feigao2016@jxstnu.com.cn (F. Gao); xlanggao@postech.ac.kr (XL. Gao)

### Content

1. Solvent swelling ........ 2

2. Calculating the yield of TMPTAA and PETMPA by $^{1}$H NMR ........ 2

  2.1 Calculating the yield of trimethylolpropane tris(3-mercaptopropionate) acetoacetate (TMPTAA) by $^{1}$H NMR ........ 2

  2.2 Calculating the yield of pentaerythritol tetra(3-mercaptopropionate) acetoacetate (PETMPA) by $^{1}$H NMR ........ 3

3. $^{1}$H NMR and $^{13}$C NMR Spectra ........ 3

## 1. Solvent swelling

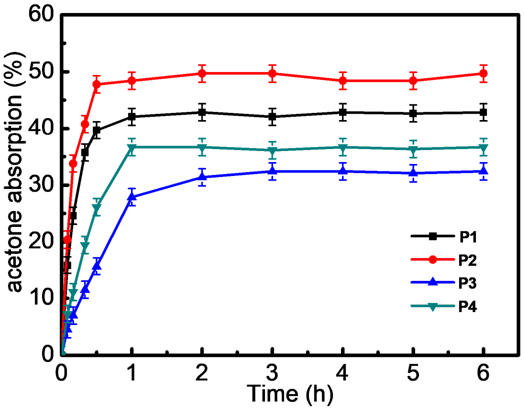


**Figure S1.** Solvent swelling (acetone absorption-time diagram) of P1, P2, P3 and P4.

## 2. Calculating the yield of TMPTAA and PETMPA by $^1$H NMR

### 2.1 Calculating the yield of trimethylolpropane tris(3-mercaptacetoacetate) (TMPTAA) by $^1$H NMR

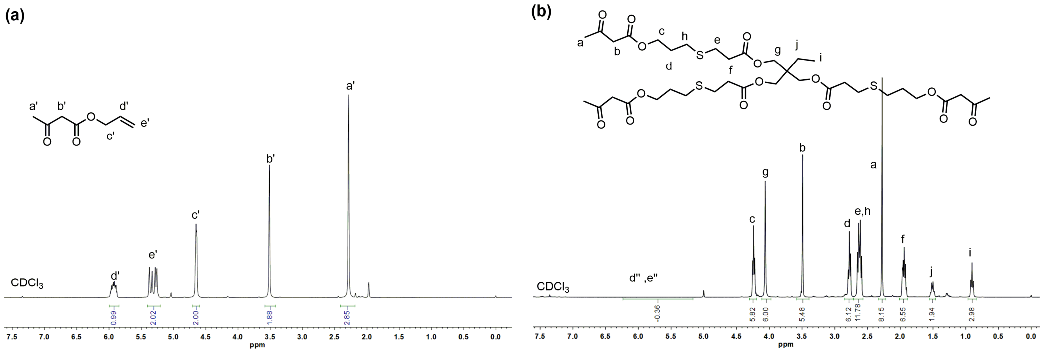


**Figure S2.** $^1$H NMR Spectrum of (a) allyl acetoacetate and (b) trimethylolpropane tris(3-mercaptacetoacetate) (TMPTAA) (400 MHz, $CDCl_3$, 7.26 ppm)

**Figure S2a** shows that the peak areas of c' is 4, and the peak area of d' is 0.99, the peak area of e' is 2.02; thus, the $^1$H NMR peak areas correspond to the compound structure. Therefore, we can calculate the conversion yield on the basis of the peak area ratios. the yield of trimethylolpropane tris(3-mercaptacetoacetate) (TMPTAA) can be calculated as: W(%) = [3×A(d' + e')-A(d'' + e'')]/[3×A(d' + e')]=[3×(0.99+2.02) - 0.36] /[3×(0.99+2.02)]= 96%.

**2.2 Calculating the yield of pentaerythritol tetra(3-mercaptacetoacetate) (PETMPA) by $^{1}$H NMR**

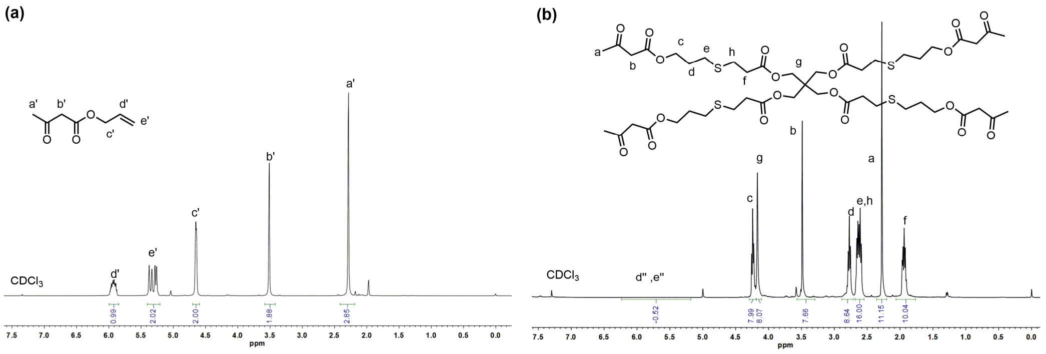


**Figure S3.** $^{1}$H NMR Spectrum of (a) allyl acetoacetate and (b) pentaerythritol tetra(3-mercaptacetoacetate) (PETMPA) (400 MHz, $CDCl_3$, 7.26 ppm)

The yield of modified cardanol is calculated using the same method as described in section 2.1: the yield of pentaerythritol tetra(3-mercaptopropionate) acetoacetate (PETMPA) can be calculated as: W(%) = [4×A(d' + e')-A(d'' + e'')]/[4×A(d' + e')]=[4×(0.99+2.02) - 0.52] /[4×(0.99+2.02)]= 93%.

## 3. $^{1}$H NMR and $^{13}$C NMR Spectra

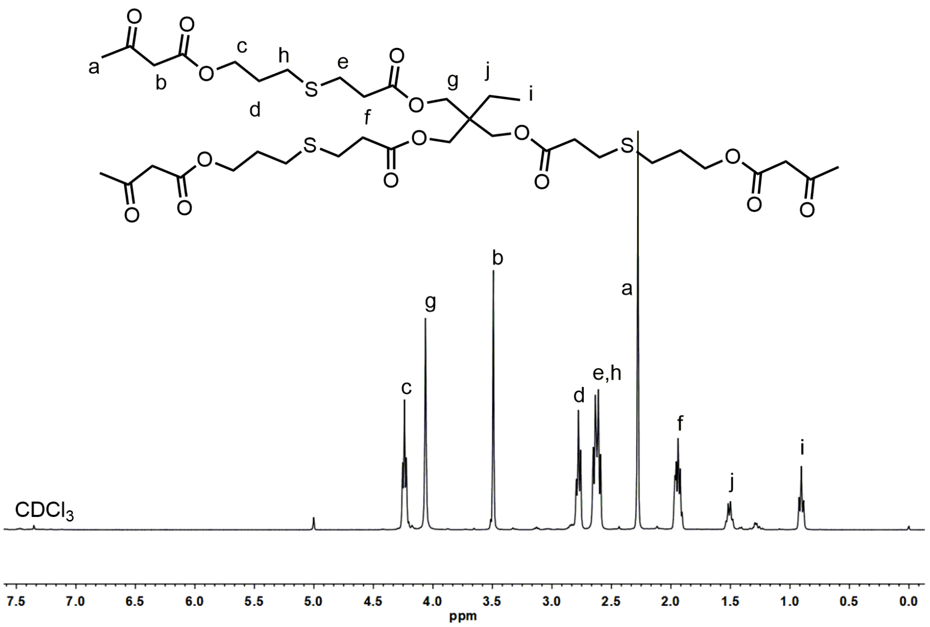

**Figure S4.** $^{1}$H NMR Spectrum of trimethylolpropane tris(3-mercaptacetoacetate) (TMPTAA) (400 MHz, $CDCl_3$, 7.26 ppm)

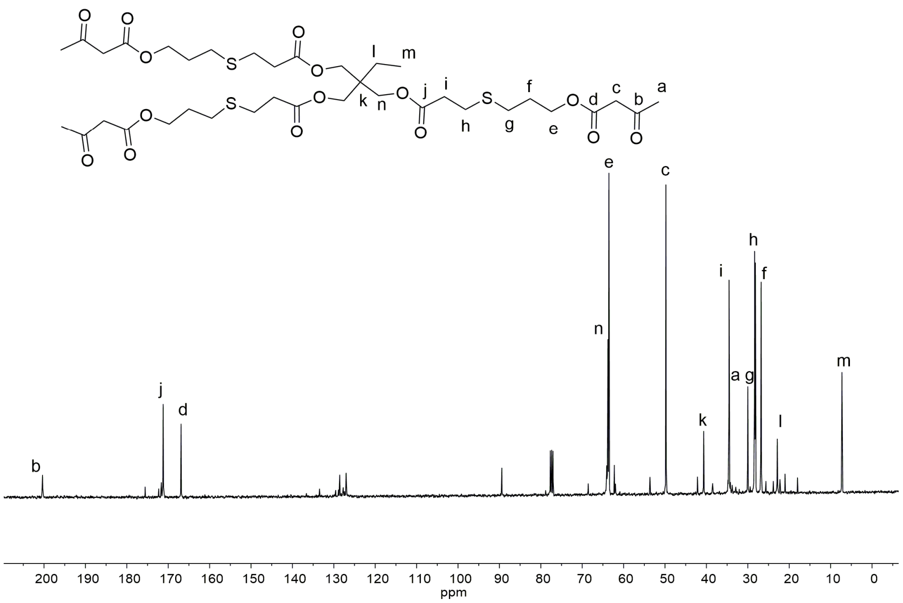


**Figure S5.** $^{13}$C NMR Spectrum of trimethylolpropane tris(3-mercaptacetoacetate) (TMPTAA) (100 MHz, $CDCl_3$, 77 ppm)

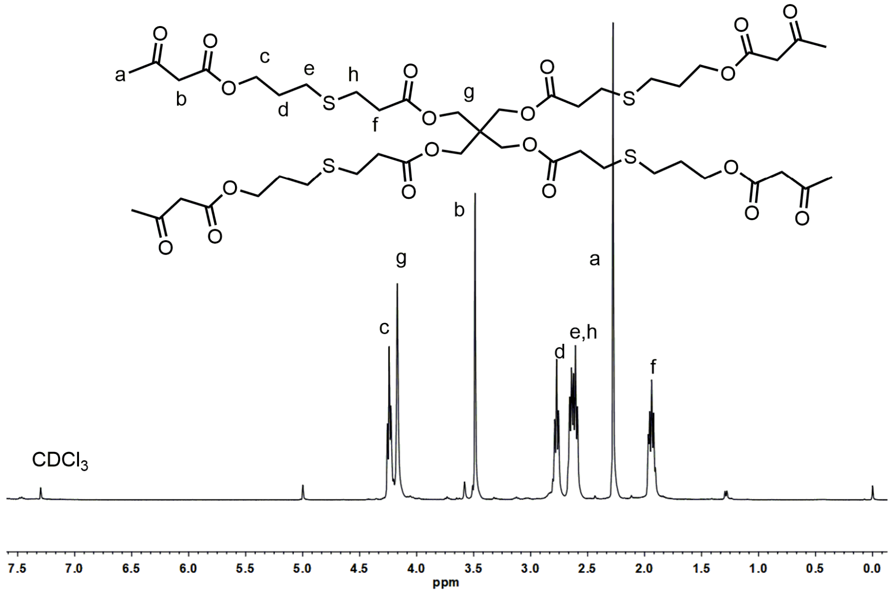


**Figure S6.** $^{1}$H NMR Spectrum of pentaerythritol tetra(3-mercaptacetoacetate) (PETMPA) (400 MHz, $CDCl_3$, 7.26 ppm)

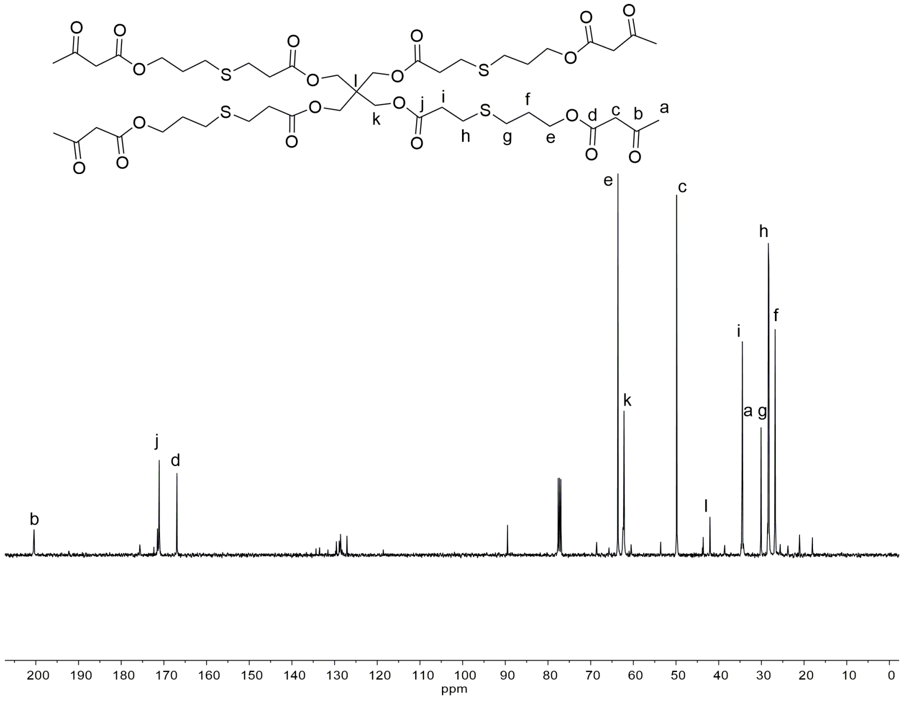


**Figure S7.** $^{13}C$ NMR Spectrum of pentaerythritol tetra(3-mercaptacetoacetate) (PETMPA) (100 MHz, $CDCl_3$, 77 ppm)
